# How Complex Dietary Fibers Can Be Used to Shape the Human Gut Microbiome Toward Reduced Inflammatory Potential: A Pilot Study

**DOI:** 10.3390/molecules31152613

**Published:** 2026-07-27

**Authors:** Maria Luisa Savo Sardaro, Sahana Kuthyar, Omolola Dada, Nimra Deivassagayame, Michael Tran, Ryder Kern, Sophie Koenig, Yosef Seidman, Matteo Atallah, Katherine R. Amato

**Affiliations:** 1 Department of Anthropology, Northwestern University, Evanston, IL 60208, USA; 2 Department of Human Science and Promotion of the Quality of Life, University of San Raffaele, 00166 Rome, Italy; 3 Division of Human Biology, Fred Hutchinson Cancer Center, Seattle, WA 98109, USA; 4 Department of Biology, Oakton College, Des Plaines, IL 60016, USA; 5 Department of Biology, New York University, New York, NY 10012, USA

**Keywords:** fiber, 16S rRNA, in vitro fermentation, SCFA, pectin, inulin, dextran, β-glucan

## Abstract

Microbiome-linked pathologies in humans have significantly increased over recent decades, suggesting that lifestyle changes, particularly those related to diet, have contributed to the disruption of beneficial microbial composition and functions. Specifically, modern processed diets that are low in dietary fiber and high in fat and sugar can lead to the depletion of bacterial taxa over generations and contribute to chronic inflammatory diseases. These pathologies can potentially be prevented by increasing fiber intake, making the promotion of dietary fiber crucial for human health. Despite the recognized importance of fiber integration, there remains a significant gap in the understanding of the use of multiple dietary fibers in food to promote microbiota diversity, as well as which dietary fibers promote specific microbial taxa to restore symbiosis. To address this gap, we conducted an in vitro fermentation study using fecal samples from two individuals. We tested three types of dietary fibers of varying complexity: inulin, pectin, and dextran in a β-glucan–based medium. Samples were collected over a 48 h fermentation period (0–4–8–24–32–48 h) to evaluate temporal shifts in microbial composition and short-chain fatty acid (SCFA) production. Through 16S rRNA gene amplicon sequencing, we found that the introduction of different fibers steered the microbiota of both individuals toward a convergent trajectory by 24–48 h. This result indicated that fiber complexity can reduce inter-individual variation in microbial community structure. Distinct levels of polysaccharide complexity between fiber types modulated specific bacterial taxa, supporting the concept that consuming a diversity of dietary fiber acts on complementary microbial niches. Notably, the observed shifts toward butyrate-associated taxa and reduction of pro-inflammatory lineages with dextran/β-glucan are relevant for pathologies characterized by dysbiosis, such as inflammatory bowel disease (IBD). Together, these results underscore the value of incorporating multiple fibers into food production, including fermented foods, to enhance prebiotic properties, stimulate the growth of fiber-fermenting bacteria, and promote microbial diversity. While these observations derive from a controlled in vitro pilot setting, they support the concept that multi-fiber dietary strategies based on complementary fermentable fibers with prebiotic properties may help shift the microbiome away from a pro-inflammatory state. Accordingly, dietary guidelines and public health approaches aimed at reducing chronic disease risk may benefit from emphasizing the inclusion of composite fiber blends rather than relying solely on single-fiber supplementation.

## 1. Introduction

The widespread epidemic of food-related disorders in recent decades, particularly in industrialized populations with Western-style diets, necessitates a careful investigation of the relationship between dietary practices and chronic diseases (CDs) [1,2,3,4,5]. For example, obesity amongst adults in the United States has shifted from 30.5% in 1999–2000 to 41.9% in 2017–2020 [6,7], with an increased forecast to 50–65% in adults by 2050 [8]. The Western diet is typically characterized by highly processed foods rich in sugars and saturated fats, and low in dietary fiber [9]. These dietary characteristics are widely believed to be responsible for the increased rates of chronic diseases such as obesity, diabetes, and cardiovascular diseases [10,11,12,13,14,15,16,17].

The increased consumption of products with high dietary sugar and low fiber content has also been shown to be associated with variation in gut microbiome composition, including a loss of biodiversity, and shifts toward a pro-inflammatory microbial profile [18,19,20,21,22,23,24,25,26,27,28,29,30,31,32]. Reduced fiber intake leads to detrimental alterations of the gut microbiota [33] with a reduction in metabolites such as short-chain fatty acids (SCFAs: acetate, propionate, and butyrate) that have a beneficial effect on intestinal gut barrier integrity and immune responses [34,35]. Low-fiber diets also alter vitamin B availability and secondary bile acid production [36]. Alongside this, high sugar intake has been linked to gut dysbiosis by increasing the relative abundances of fast-growing Proteobacteria compared to slower-growing commensals involved in the breakdown of complex carbohydrates. These dynamics reduce microbial diversity, disrupt microbiota balance, and promote pro-inflammatory conditions by weakening epithelial integrity and mucosal immunity [28,37]. Elevated Proteobacteria abundance has been previously associated with inflammatory conditions such as inflammatory bowel disease (IBS), asthma, chronic obstructive pulmonary disease, cardiovascular diseases [38], and IBD [39], as well as increased susceptibility to pathogen infections [40].

Dietary strategies intended to achieve health benefits by increasing gut microbiota diversity advocate for the consumption of complex non-digestible substrates and substrate mixtures [41,42]. For instance, Liu et al. (2024) reported that a 5% increase in the intake of complex insoluble dietary fibers (IDF) was effective in suppressing weight gain induced by a high-fat diet [43]. However, dietary fibers differ considerably in their structural characteristics, and each has been shown to shape the gut microbiome and impact host health in distinct ways. Dietary fibers such as inulin, β-glucan with mannans, pectin, and dextran can differentially promote the growth of beneficial bacteria according to their structural complexity [44]. In the present study, these fibers were selected to represent a gradient of structural complexity, with inulin representing the least structurally complex substrate and dextran the most structurally complex.

Inulin, a fructose oligosaccharide, is one of the most common fibers used in food production. Inulin has been shown to act as a stimulus for the growth of beneficial *Bifidobacterium* [45,46,47] and exhibits anti-inflammatory properties in colitis by modulating the gut microbiota and reinforcing gut barrier integrity in mice [48]. In tumor-bearing mice, an inulin gel formulation normalized the dysregulation of the gut microbiome, improving host immune responses and decreasing immune checkpoint inhibitors associated with colitis [49]. It has also been shown to reduce the progression of chronic kidney disease in a mouse model [50]. However, there are also some conflicting results in that inulin has been reported to potentially exacerbate intestinal damage [51] and chronic inflammation in mouse models [40,51].

Baker’s yeast β-glucan (YBG) is a non-digestible complex polysaccharide (β-1,3/1,6-D-glucan) that significantly increases the amount of polysaccharide-fermenting bacteria, such as Bacteroidetes and Verrucomicrobia, in mouse models [52,53,54,55,56,57]. Baker’s yeast extract also contains mannans with an α-1,6-linked backbone and α-1,2- and α-1,3-linked mannose branches [58], polysaccharides that promote Bacteroidetes growth [59,60,61].

Pectin is a soluble fiber that is shown to exert protective effects against inflammatory disorders like colitis, promoting the growth of beneficial bacteria such as *Lactobacillus* and *Bifidobacterium* [62], affecting immunoregulatory pathways [63] and reducing gut inflammation in pigs [64,65] and mice [66]. Similarly, studies have shown that consumption of pectin-rich baobab fruit pulp powder increases the relative abundances of typically healthy gut microbial taxa including Bifidobacteria, Lactobacilli, and Firmicutes [67,68].

Dextran fiber is a high molecular weight food-grade homopolysaccharide of D-glucose produced by the lactic acid bacteria *Weissella cibaria*. Early in vitro studies on the human gut microbiome showed that dextran reduced pH and increased the generation of SCFAs such as acetate and propionate [69]. It is one of the most complex dietary fibers (DFs) with the potential to positively modulate the gut microbiota [47], promoting increased relative abundances of *Bacteroides* and lower gas production compared to inulin in humans [70].

Despite this rich body of literature, knowledge gaps remain regarding the impact of fiber complexity on bacterial succession and metabolic recovery during fermentation. Additionally, while there is growing evidence of the benefits of individual fibers on microbiome composition and function, the combined effects of multiple fibers with differing molecular structures remain poorly characterized. To address these gaps, this study uses an in vitro fermentation model to explore how the different complexities of dietary fiber polysaccharides can shape gut microbiome composition by examining the effects of inulin, pectin, and dextran fibers combined with β-glucans and mannans from baker’s yeast extract. In vitro fermentation models provide a controlled framework to explore these interactions by simulating gut-like anaerobic conditions and tracking community-level responses over time. This approach allows us to disentangle how specific polysaccharides and their combinations shape microbial adaptation and functional balance. Specifically, we hypothesized that the level of fiber complexity would influence fermentability and the bacterial groups able to utilize them. We predicted that less complex fibers would be more accessible to fast-growing species, while more complex polysaccharides might favor bacteria with specialized enzymatic capacities. We also aimed to understand the extent to which combining fibers enhances microbial diversity and functional resilience beyond what is observed with single fibers, with potential applications for restoring gut homeostasis. Ultimately, this work aims to provide a possible approach for the development of fiber-enriched and fermented foods, as well as the design of precision nutritional and microbiome-based therapeutic interventions to counteract dysbiosis and chronic inflammation.

## 2. Results

**Figure 1:**
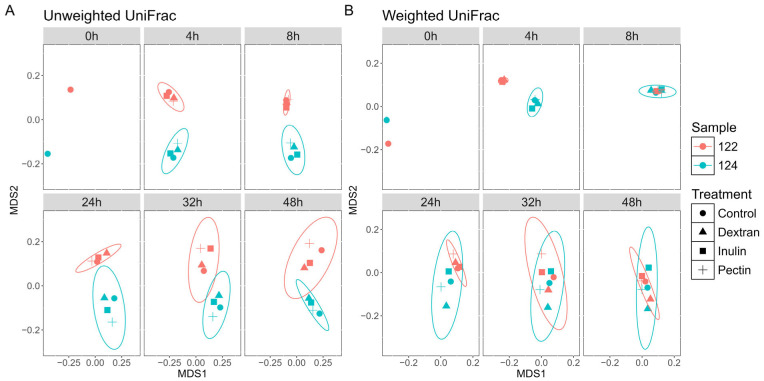
Non-metric multidimensional scaling (NMDS) plots based on unweighted UniFrac (**A**) and weighted UniFrac (**B**) distances of two fermented fecal microbial communities from two healthy donors (122 and 124) during in vitro fermentation. Samples from the control (β-glucans-mannans), dextran (control supplemented with dextran), inulin (control supplemented with inulin), and pectin (control supplemented with pectin) treatments were collected at 0, 4, 8, 24, 32, and 48 h. Ellipses indicate the clustering of samples from each donor.

Beta diversity assessments using unweighted and weighted UniFrac distances (Figure 1A,B) revealed notable changes in microbial community composition over time and between treatments. Significant differences in the presence/absence of bacterial taxa and the abundance of common species were observed between the two donor individuals (unweighted UniFrac: F = 7.27, R = 0.12, *p* = 0.002: weighted UniFrac: F = 3.03, R = 0.69, *p* = 0.0002) and across all collection time points (unweighted UniFrac: F = 1.46, R = 0.51, *p* = 0.003: weighted UniFrac: F = 6.22, R = 0.071, *p* = 0.003). Notably, the compositional dissimilarity between the two donor individuals decreased after 24 h of fermentation, with the clusters becoming more closely aligned in ordination space. However, this pattern began to emerge after as little as 8 h. In the unweighted UniFrac analysis, inter-individual distances based on the presence or absence of taxa were reduced by 8 h, showing progressive convergence at 24–48 h (Figure 1A). In contrast, the weighted UniFrac analysis revealed an early increase in similarity within the first 8 h in terms of the relative abundance of shared taxa, followed by greater divergence at later time points, corresponding to fiber-specific differences in the abundance of common taxa between donors (Figure 1B). At 48 h, the two clusters substantially overlapped, with samples from both donor individuals with the same fiber positioned in close proximity to one another, demonstrating the capacity of each fiber to promote a specific group of bacteria even in microbial communities that are initially distinct.

**Figure 2:**
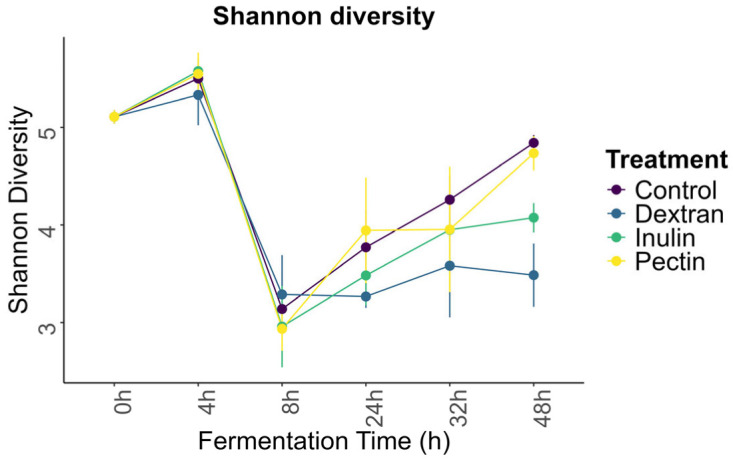
Shannon alpha diversity of the fecal microbial communities during in vitro fermentation across the control (β-glucans-mannans), dextran (control supplemented with dextran), inulin (control supplemented with inulin), and pectin (control supplemented with pectin) treatments at 0, 4, 8, 24, 32, 48 h.

We observed a significant decline in microbial diversity, measured by the Shannon Index, at 8 h across all treatments, with dextran supplementation exhibiting a smaller decline compared to the other treatments (4–8 h: Control *p* = 0.003; Inulin *p* = 0.001; Pectin *p* = 0.001; Dextran *p* = 0.01; Figure 2). After 8 h, a sharp recovery in microbial diversity was observed in the control and pectin samples, followed by inulin, while dextran fermentation showed a slower increase in diversity between 24–48 h. A similar pattern was also reflected in the observed amplicon sequence variants (ASVs) and in Faith PD indices (Figures S1 and S2 respectively in Supplementary Materials).

**Figure 3:**
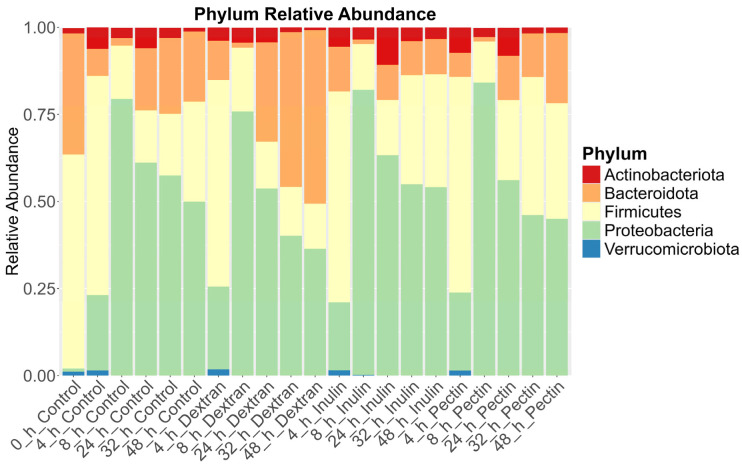
Relative abundances of bacterial phyla in the combined fecal microbial communities from two healthy donors (122–124) during in vitro fermentation. Samples from the control (β-glucans-mannans), dextran (control supplemented with dextran), inulin (control supplemented with inulin), and pectin (control supplemented with pectin) treatments were collected at 0, 4, 8, 24, 32, 48 h.

Irrespective of changes in overall composition, the gut microbiome of both donor individuals exhibited clear shifts in the relative abundances of phylum-level taxa in response to fiber interventions and associated sugar content (Figure 3). Early fermentation stages (4 h) showed a transient increase of alpha diversity in Figure 2, Figures S1 and S2. This was associated with a rapid growth of Proteobacteria, which exploit the readily available sucrose in the medium, reaching a relative abundance of 25% in all treatments (Figure 3). By 8 h, this expansion resulted in decreased evenness and phylogenetic diversity, reflecting dominance by a few fast-growing taxa still associated with Proteobacteria phylum. After 8 h, microbial diversity began to recover, coinciding with the rise of fiber-utilizing bacterial groups like Firmicutes and Bacteroidota. These changes in relative abundance at the phylum level also explain the α-diversity fluctuations observed in the Shannon Index (Figure 2).

Regression analysis at the phyla level revealed a slight decrease in the relative abundance of Firmicutes at 4 h in all samples, reaching the lowest relative abundances and statistical significance at 8 h (0–8 h: Control–Control, *p* = 0.008; Control–Inulin, *p* = 0.005; Control–Pectin, *p* = 0.004; Control–Dextran, *p* = 0.01) (Figure 3). A similar pattern was observed for Bacteroidota (0–8 h: Control–Control, *p* = 0.006; Control–Inulin, *p* = 0.005; Control–Pectin, *p* = 0.005; Control–Dextran, *p* = 0.005). In contrast, Proteobacteria exhibited a strong and significant increase in relative abundance over the same time period (0–8 h: Control–Control, *p* = 0.0001; Control–Inulin, *p* = 0.00008; Control–Pectin, *p* = 0.00006; Control–Dextran, *p* = 0.0002), likely driven by the 0.5% of sucrose present in the medium, which may have provided a readily fermentable substrate favoring their rapid expansion. Actinobacteriota also showed a significant increase in relative abundance at 4 h in all samples (*p* = 0.05), followed by a reduction at 8 h and a modest rebound at 24 h, particularly in the inulin and pectin treatments (0 h Control–24 h Inulin, *p* = 0.01; 8 h Control–24 h Inulin *p* = 0.025; 0 h Control–24 h Pectin, *p* = 0.06) (Figure 3). Dextran samples showed a different pattern with a consistent increase of Bacteroidota relative abundance at 24–48 h compared to the control samples at 8 h (Dextran vs. Control: 24 h *p* = 0.02; 32 h *p* = 0.0006; 48 h *p* = 0.0001), accompanied by a reduction in Proteobacteria (Dextran vs. Control: 24 h *p* = 0.14; 32 h *p* = 0.03; 48 h *p* = 0.01) and a slight but significant decrease in Firmicutes relative abundances (Dextran vs. Control: 24–4 h *p* = 0.003; 32–4 h *p* = 0.003; 48–4 h *p* = 0.002). A slight trend of increasing Bacteroidota relative abundance was also observed in the control samples, approaching significance at 32 h (Control 8–32 h *p* = 0.07). Collectively, these results indicate that the early sucrose-driven bloom of Proteobacteria was associated with the reduction in α-diversity, while the subsequent fiber fermentation progression selectively restored community evenness and phylogenetic diversity in a fiber-specific manner.

**Figure 4:**
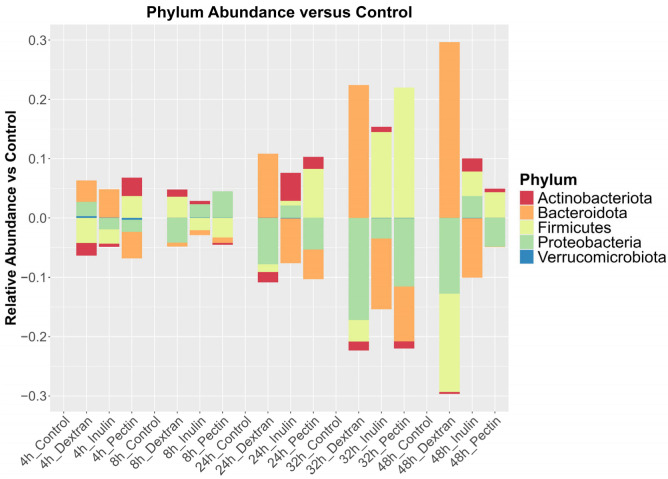
Fiber-specific differences in bacteria phyla visualized by differential abundance analysis using the control samples as the reference calibrator. Relative abundances of bacteria phyla in the combined fecal microbial communities from two healthy donors (122–124) are shown for the control (β-glucans-mannans), dextran (control supplemented with dextran), inulin (control supplemented with inulin) and pectin (control supplemented with pectin) treatments at 0, 4, 8, 24, 32, 48 h.

The fiber-specific phylum-level differences become more evident when visualized as differential abundance comparisons at the phylum level, using the control samples as a reference (Figure 4). Here, the relative abundance of each taxon in the control samples was subtracted from that of the fiber-supplemented samples, thereby isolating changes specifically attributable to the added fibers. Consistent with the overall phylum-level changes in Figure 3, Bacteroidota displayed an incremental trend in the Dextran samples, accompanied by a greater suppression of Proteobacteria relative to the control from 8 h to 48 h. Actinobacteriota relative abundance increased with inulin supplementation at 24 h, concurrent with a reduction in Bacteroidota relative abundance and less effective control of Proteobacteria compared with the control treatment. Pectin supplementation was associated with increases in both Firmicutes and Actinobacteriota at 24 h.

Overall, these results show that distinct fiber substrates differentially modulated the sucrose-driven proliferation of Proteobacteria to varying degrees. The fiber substrates also selectively promoted the recovery or enrichment of specific phyla including Actinobacteriota, Bacteroidota and Firmicutes, resulting in distinct fiber-specific fermentation trajectories.

**Figure 5:**
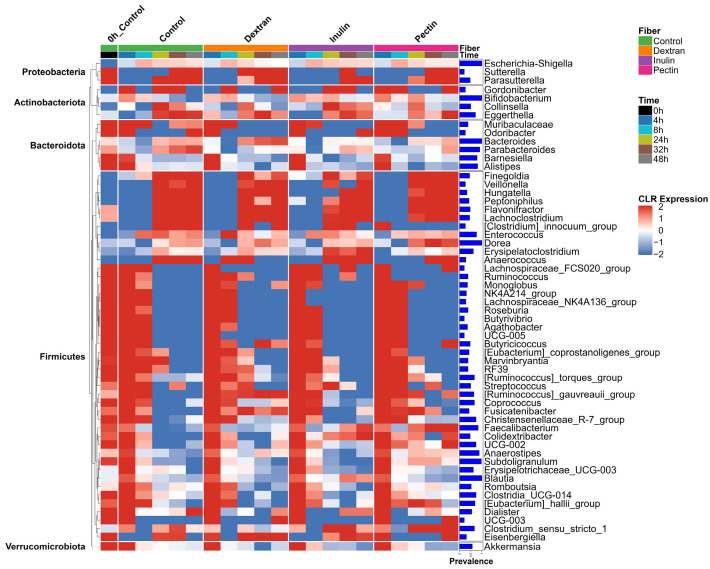
Genus-level microbial dynamics across fiber treatments during in vitro fermentation. CLR-transformed relative abundances of bacterial genera in the combined fecal microbial communities from two healthy donors (122–124) are shown for the control (β-glucans-mannans), dextran (control supplemented with dextran), inulin (control supplemented with inulin) and pectin (control supplemented with pectin) treatments at 0, 4, 8, 24, 32, and 48 h. Rows represent bacterial genera grouped by phylum, and columns represent time points within each fiber treatment. Color intensity indicates centered log-ratio (CLR) relative abundance (red = higher relative abundance; blue = lower relative abundance).

To determine whether phylum-level shifts were driven by distinct bacterial taxa, we examined the CLR-transformed heatmap, which illustrates genus-level relative abundances across fermentation time points and fiber treatments (Figure 5). Early fermentation stages (0–8 h) were initially dominated by Firmicutes, followed by an expansion of inflammatory Proteobacteria, particularly *Escherichia*–*Shigella* at 8 h (ANCOM-BC q-value < 0.005) across all treatments. This expansion coincided with the transient decline in α-diversity observed during the early stages of fermentation (Figure 2). As fermentation progressed, these opportunistic taxa declined in relative abundance, while fiber-associated fermenters expanded in a substrate-specific manner. For example, *Bacteroides* relative abundances increased in the dextran treatment. Nevertheless, not all taxa were able to recover in response to fiber. For example, the relative abundance of *Eubacterium coprostanoligenes*, a mucin-associated bacteria important for the integrity of the intestinal mucus barrier [71], did not recover.

Across all fiber treatments, several taxa increased in relative abundance after 8 h. These included *Finegoldia* (ANCOM-BC q-value < 0.005 at 32 h), *Veillonella* (q-value < 0.01), *Hungatella* (q-value < 0.005 at 48 h), *Peptoniphilus* (q-value < 0.02 at 32 h), *Flavonifractor* (q-value < 0.005 at 48 h), *Lachnoclostridium* (q-value < 0.005 at 48 h), and *Eggerthella* (q-value < 0.005 from 24 to 48 h), indicating a common temporal response across treatments. *Clostridium innocuum*_*group* also increased in relative abundance, occurring at 48 h in the control and dextran treatments and 24 and 32 h in the inulin treatment. Similarly, *Eisenbergiella* increased across multiple treatments, occurring at 24 and 32 h in the control, at 4, 24, 32, and 48 h in dextran and inulin, and at 4 and 48 h in pectin.

In the control treatment (β-glucans/mannans), *Parabacteroides* showed an elevated relative abundance at 32–48 h (ANCOM-BC q-value < 0.002) followed by a modest increase in the relative abundances of *Bacteroides* at 32–48 h (q-value = 0.0056 at 48 h) and *Muribaculaceae* at 32–48 h (q-value < 0.005). Most Firmicutes displayed low CLR values, except for the *Clostridium_innocuum_group* at 48 h (q-value < 0.005), indicating limited recovery of saccharolytic and only partial rebalancing supported by β-glucans/mannans alone. Relative abundances of *Ruminococcus_torques_group* and *Ruminococcus_gauvreauii_group* decreased from 24 to 48 h (q-value < 0.005). In contrast, *Clostridium sensu stricto_1* increased at 32–48 h (q-value < 0.005) while *Anaerococcus* relative abundances increased from 24 h onward (q-value < 0.005 at 32 h). *Escherichia–Shigella* remained abundant throughout the fermentation period (8–48 h; q-value < 0.005). We also observed a reduction in the relative abundance of *Faecalibacterium* from 24 to 48 h (q-value < 0.005), and *Butyrococcus* decreased from 24 to 48 h (q-value < 0.005).

The inulin treatment favored the proliferation of *Bifidobacterium* at 8 h (ANCOM-BC q-value < 0.04), with a weaker growth persisting until 24 h (q-value < 0.1). Subsequently, *Collinsella* increased in relative abundance from 24 to 48 h (q-value < 0.005 at 24 h). The inulin treatment also enriched *Parabacteroides* at 32–48 h (q-value < 0.01) and butyrate-producing Firmicutes, including *Faecalibacterium* at 32–48 h (q-value < 0.1). In addition, *Colidextribacter* showed a non-significant increase in relative abundance (q-value = 0.3 at 48 h). Similarly, *Erysipelatoclostridium* and *Blautia* showed non-significant increases from 24 to 48 h (q-values > 0.4). Increased relative abundances were also observed for *Clostridium_sensu_stricto*_*1* from 24 h onward (q-value < 0.005) and *Anaerococcus* from 24 to 48 h (q-value < 0.001), whereas *Eisenbergiella* showed an increasing trend that was not statistically significant (q-value > 0.8).

Pectin supplementation also induced significant genus-level shifts over time. *Faecalibacterium* increased in relative abundance at 24–48 h (ANCOM-BC q-value < 0.05 at 32 h) [72]. *Eubacterium_hallii_group*, a known butyrate and propionate producer [73,74,75], maintained elevated relative abundance until 32 h (q-value = 0.007 at 32 h), while *UCG-002* remained significantly elevated through 48 h (q-value < 0.001 at 32–48 h). *UCG-003* showed an increasing trend at 48 h, although it did not reach statistical significance. *Colidextribacter* increased in relative abundance at 24 h and remained elevated at 48 h (q-value < 0.05 at 24 h) [76]. Partial maintenance of butyrate-associated taxa, including *Subdoligranulum* (q-value = 0.01 at 48 h) [77] and *Anaerostipes* was observed from 24 to 48 h (q-value < 0.005 at 48 h) [78]. Although *Bifidobacterium* displayed elevated CLR values in the heatmap up to 24 h, this trend did not remain significant after FDR correction (q-value = 0.1). A reduction of *Escherichia–Shigella* was observed at 24 h (q-value < 0.005). In parallel, *Ruminococcus_torques_group* remained at elevated relative abundance until 32 h before decreasing at 48 h (q-value < 0.005 at 24–48 h) [79]. *Anaerococcus* increased in relative abundance from 32 to 48 h (q-value = 0.03 at 32 h), while *Clostridium_sensu_stricto_1* increased from 24 h onward, reaching strong significance at 32 h (q-value < 0.005).

Dextran supplementation resulted in marked enrichment of *Bacteroides* at 32 and 48 h (ANCOM-BC q-value < 0.000018). It was accompanied by increased relative abundances of *Butyricicoccus* (q-value < 0.005 for all time points) and *Ruminococcus_gauvreauii_group* (q-value < 0.005 for all time points), together with reduced CLR values for *Ruminococcus_torques_group* at 32–48 h (q-value < 0.005). *Fusicatenibacter* [80] increased in relative abundance at 4 h (q-value < 0.005), and maintained high CLR values throughout the remaining time points. *UCG-002* started to decrease at 8 h and increased again by 48 h (q-value < 0.005), whereas *UCG-003* follow a similar trend but did not reach statistical significance (q-value = 0.29). *Clostridium_innocuum_group* increased in relative abundance at 32–48 h (q-value < 0.002) while *Eisenbergiella* increased from 24 to 48 h (q-value < 0.005 at 48 h). Pronounced reductions were also observed for *Escherichia–Shigella* from 24 to 48 h (q-value < 0.005), *Streptococcus* at 4 h (q-value < 0.005) and *Anaerococcus* from 32 to 48 h (q-value < 0.005).

**Figure 6:**
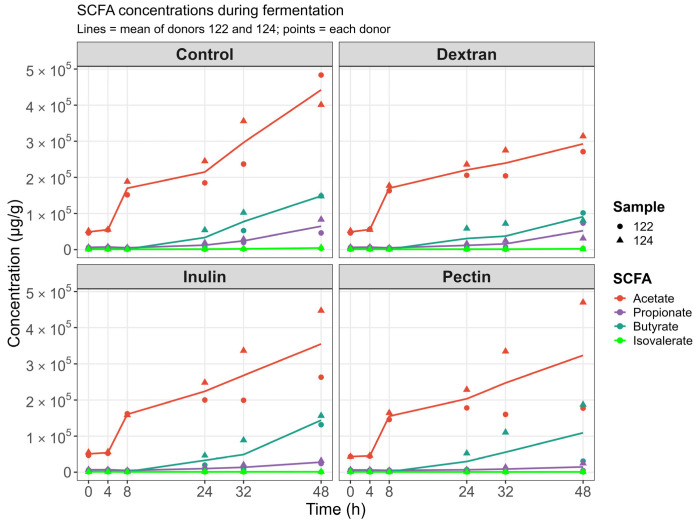
Short-chain fatty acid (SCFA) concentrations (µg/g) in the combined fecal microbial communities from two healthy donors (122 and 124) during in vitro fermentation. Samples from the control (β-glucans-mannans), dextran (control supplemented with dextran), inulin (control supplemented with inulin), and pectin (control supplemented with pectin) treatments were collected at 0, 4, 8, 24, 32, and 48 h. Lines represent the mean SCFA concentration of donors 122 and 124, whereas points represent individual donor measurements (circle = donor 122; triangle = donor 124).

Short-chain fatty acid (SCFA) profiling revealed substrate-dependent patterns in both the magnitude and proportionality of fermentation products (Figure 6). The β-glucan/mannan control condition produced the highest acetate levels, consistent with rapid fermentation by *Parabacteroides* and other saccharolytic taxa. This treatment showed strong time effects for acetate, butyrate, and propionate (Acetate: F = 19.9, *p* = 0.003; Butyrate: F = 24.8, *p* = 0.002; Propionate: F = 10.4, *p* = 0.011), confirming its robust fermentative potential. However, across the whole experiment, there was an increase in acetate over butyrate.

Inulin produced a coordinated SCFA response, with significant time effects for acetate (*p* = 0.014), butyrate (*p* = 0.007), and propionate (*p* = 0.014). The gradual and parallel increases across all SCFAs reflect broad activation of saccharolytic and cross-feeding pathways, consistent with inulin’s well-established fermentability.

Pectin showed the strongest donor-specific divergence. Donor 124 generated substantially higher acetate and butyrate concentrations than donor 122, resulting in high variance and the absence of significant time effects (all *p* > 0.09) despite similar trends at later stages. This suggests that pectin fermentation is highly dependent on baseline microbiota composition and underscores the need for larger donor cohorts when evaluating pectin responsiveness.

Dextran supplementation yielded a more balanced acetate–butyrate profile. Acetate concentrations increased significantly (F = 55.8, *p* = 0.0002) but at lower levels than in the control, while butyrate and propionate did not reach significance (*p* = 0.085 and *p* = 0.123). This pattern aligns with the moderate increase of butyrate-producing Firmicutes relative abundance, high Bacteroidota relative abundance, and reduced Proteobacteria relative abundance observed in the microbiome data. This is an important pattern to consider since the concomitant presence of β-glucan and dextran led to the increased relative abundance of the Bacteroidota plylum and, in particular, *Bacteroides* sp.

## 3. Discussion

In this study, we used batch incubation of gut microbial communities with multiple fibers to determine how structurally distinct dietary fibers shape microbiome composition and metabolic outputs over time. The combined analysis of alpha and beta diversity, relative abundances of individual taxa, and SCFA profiles reveal a substrate-dependent ecological succession during fermentation that converges over time in the two individuals. Overall, our findings suggest that fiber structure drives distinct microbial and metabolic trajectories across successive fermentation stages, with specific fibers promoting complementary microbial functions and cross-feeding interactions that influence community balance and SCFA metabolism. Together, these observations support the concept that combining structurally distinct fibers may provide complementary ecological benefits that cannot be achieved through single-fiber supplementation alone.

Across all treatments, early time points were characterized by a transient expansion of Proteobacteria, particularly *Escherichia–Shigella*, coinciding with the decline in Shannon diversity and Faith’s PD. This sucrose-driven bloom temporarily reduced community evenness and phylogenetic breadth [28]. However, as simple carbohydrates were depleted, fiber-specific fermentative networks progressively reshaped community composition, leading to convergence between donors by 24–48 h. This convergence indicates that fiber structure exerts a stronger ecological force than baseline inter-individual variability under controlled fermentation conditions.

At the phylum level, differential recovery of Firmicutes, Bacteroidota, and Actinobacteriota corresponded closely to the genus-level restructuring observed across treatments, while all fibers mitigated the early Proteobacteria bloom through distinct ecological trajectories. The control samples were characterized by an increase of Firmicutes and Bacteroidota. Inulin preferentially stimulated Actinobacteriota, whereas pectin promoted a broader recovery of Firmicutes and Actinobacteriota, but with greater donor-dependent variability. Dextran was characterized by a progressive enrichment of Bacteroidota together with the most consistent reduction of Proteobacteria. These phylum-level differences were reflected by the enrichment of distinct fermentative taxa and ultimately contributed to the fiber-specific SCFA profiles observed during fermentation.

In the β-glucan/mannan control condition, the enrichment of *Parabacteroides* [81] and a modest expansion of *Bacteroides* indicate effective activation of saccharolytic Bacteroidota pathways. This is consistent with the strong acetate-producing baseline observed in SCFA profiling [82]. Maintenance of *Muribaculaceae* suggests partial engagement of carbohydrate-degrading and cross-feeding networks [83]. However, most Firmicutes remained low, and key butyrate-associated taxa including *Faecalibacterium* and *Butyricicoccus* were reduced, indicating limited downstream conversion of acetate into butyrate [84,85]. In parallel, persistent enrichment of *Escherichia*–*Shigella* and increased abundance of *Clostridium sensu stricto_1* and *Anaerococcus* [86] suggest incomplete suppression of opportunistic taxa. *Escherichia–Shigella* and *Clostridium sensu stricto_1* are associated with ulcerative colitis and with *Clostridium difficile* infection, respectively, and are considered potentially harmful or pro-inflammatory organisms [87,88,89]. Members of the *Escherichia–Shigella* group, including adherent-invasive *Escherichia coli* (AIEC), have been shown to induce Th17 responses, enhance Th1 cell accumulation, and promote pro-inflammatory cytokine production in Crohn’s disease and fibrotic progression [90,91,92]. Moreover, decreased abundances of *Butyricicoccus* have been reported in several inflammatory disorders, including IBS and kidney disease [93,94,95]. Although reduction of the *Ruminococcus_torques_group* may reflect decreased mucin degradation, the overall ecological shift remained partial. These findings suggest that β-glucans and mannans establish strong fermentative activity but do not fully restore ecological balance or promote robust butyrate-producing consortia. This highlights the limitations of single-fiber stimulation in achieving complete microbial restructuring.

Inulin elicited a classical, coordinated prebiotic restructuring of the microbial ecosystem. Early enrichment of *Bifidobacterium* was observed, followed by sustained expansion of other Actinobacteriota such as *Collinsella* during later fermentation stages. This pattern is consistent with the well-established specialization of bifidobacteria for fructan metabolism, which generates acetate and lactate as primary end products [96,97]. Although not all increases remained significant after multiple-testing correction, the overall trajectory supports selective activation of Actinobacteriota-driven saccharolysis. As fermentation progressed, inulin supplementation was accompanied by an expansion of *Parabacteroides* and butyrate-associated Firmicutes, including *Faecalibacterium*. This suggests an engagement of cross-feeding interactions in which acetate and lactate derived from bifidobacterial metabolism are secondarily converted into butyrate [72,80,98]. This distributed activation of primary degraders and secondary fermenters corresponded with the coordinated increase observed in acetate, propionate, and butyrate, indicating balanced fermentative output rather than dominance of a single metabolic pathway. Additional genera, including *Colidextribacter*, *Blautia*, and *Erysipelatoclostridium*, exhibited increasing trends, reflecting broader activation of saccharolytic networks [76]. However, concomitant increases in *Clostridium_sensu_stricto_1*, *Anaerococcus*, and *Eisenbergiella* suggest that inulin fermentation may also permit expansion of certain opportunistic pathogens at later stages. Thus, while inulin promoted enrichment of health-associated saccharolytic and butyrate-producing consortia, its ecological effects appear more specialized and metabolically focused and comparatively less effective in suppressing pro-inflammatory taxa than those observed under dextran supplementation.

Pectin supplementation induced substantial genus-level restructuring, but with the highest donor-dependent variability among treatments. Enrichment of butyrate-associated taxa, including *Faecalibacterium*, *Eubacterium hallii_group*, *Subdoligranulum*, and *Anaerostipes*, indicates activation of secondary fermentative pathways during later fermentation stages [74,75,77,78,99,100]. Sustained elevation of *UCG-002* and transient increases in *Colidextribacter* [76] further support the engagement of saccharolytic networks. Although *Bifidobacterium* displayed elevated relative abundance in early heatmap patterns, this trend did not remain significant after FDR correction, suggesting that pectin does not consistently drive classical Actinobacteriota-dominated prebiotic responses. Concomitant ecological shifts complicate this interpretation. Increases in *Clostridium_sensu_stricto_1* and *Anaerococcus*, together with delayed modulation of *Ruminococcus_torques_group* [79], indicate that pectin fermentation supports a mixed microbial response rather than a uniformly beneficial restructuring. While *Escherichia–Shigella* declined at intermediate time points, suppression of opportunistic taxa was less consistent compared with dextran. Functionally, SCFA trajectories under pectin showed upward trends in acetate and butyrate; however, marked divergence between donors resulted in high variance and a lack of statistical consistency over time. This heterogeneity suggests that pectin fermentation is strongly dependent on baseline microbiota composition, likely reflecting inter-individual differences in polysaccharide utilization loci and enzymatic capacity for complex homogalacturonan degradation.

Dextran supplementation produced a coordinated restructuring of the microbial community. The enrichment of *Bacteroides* suggests strong activation of α-1,6–linked glucan-degrading pathways, consistent with the broad polysaccharide utilization capacity of this genus [101]. Although the primary fermentation end products of Bacteroidota species are acetate and propionate, these organisms are recognized as key degraders of complex polysaccharides and indirectly promote butyrate production through cross-feeding interactions with butyrate-producing Firmicutes [84,101]. Unlike the control condition, dextran was accompanied by expansion of multiple butyrate-associated Firmicutes, including *Butyricicoccus*, *Fusicatenibacter*, and *Ruminococcus_gauvreauii_group*, indicating enhanced cross-feeding networks, linking primary degraders to secondary fermenters [84,97,101]. The reduction of *Ruminococcus_torques_group* further suggests decreased reliance on mucin-derived substrates and a shift toward fiber-driven metabolism. Beyond carbohydrate degradation, this restructuring may carry broader functional implications.

Human gut isolates of *Bacteroides* have been shown to produce γ-aminobutyric acid (GABA) across a wide concentration range. GABA is implicated in nervous system development and gut–brain axis signaling. Thus, the dextran-associated increase in Bacteroidota may extend to modulation of neuroactive metabolic outputs [102,103].

Several *Bacteroides* species also express bile salt hydrolases that deconjugate primary bile acids into secondary bile acids, reshaping the bile acid pool and contributing to colonization resistance through inhibition of pathogens such as *Clostridium difficile* [104,105,106]. In murine models, reduced abundance of *Bacteroides* accompanied by increased Firmicutes has been associated with obesity, likely due to enhanced caloric extraction efficiency under specific microbial configurations [107]. Alterations in the Bacteroidota-to-Firmicutes balance have similarly been implicated in IBS pathology [108,109].

Within this context, the dextran-associated enrichment of *Bacteroides* suggests a microbial configuration more frequently linked with metabolic and immune balance. Dextran supplementation was also associated with a pronounced suppression of opportunistic and potentially pro-inflammatory genera, including *Escherichia–Shigella*, *Streptococcus*, and *Anaerococcus*, suggesting improved ecological control compared with the control fiber alone. Their suppression may reflect an attenuation of inflammatory signaling potential. While increases in *Clostridium_innocuum_group* and *Eisenbergiella* were observed at later stages, the overall pattern reflected a transition toward saccharolytic and butyrate-supporting consortia rather than persistence of inflammatory taxa [87,88,89]. Moreover, decreased abundances of *Butyricicoccus* have been reported in several inflammatory disorders, including IBS and kidney disease [93,94,95]; further supporting a shift toward a functionally protective configuration. The overall pattern aligns also with the lower ratio of acetate/butyrate as a result of a higher conversion of acetate into butyrate. Increased butyrate production has been associated with improved epithelial barrier function, immune regulation, and protection against colorectal cancer [110,111].

Across treatments, late-stage convergence in SCFA production mirrored the convergence in the beta diversity analyses, indicating adaptive restructuring of fermentative metabolic groups. While β-glucans/mannans established a strong acetate-producing baseline, dextran, inulin and pectin supplementation promoted more proportionate fermentative outputs and a more consistent suppression of inflammatory-associated taxa associated with dextran supplementation.

Collectively, these findings demonstrate that fiber structure governs not only fermentative magnitude but ecological directionality and functional potential. Single-fiber substrates may stimulate rapid fermentation yet fail to fully suppress opportunistic taxa or restore cross-feeding stability. In contrast, specific combinations of structurally diverse polysaccharides may promote complementary microbial niches, enhance ecological resilience, and support metabolic balance and intestinal homeostasis.

The inclusion of two healthy donors in this pilot study limited our ability to fully characterize inter-individual variability in microbial responses to fiber supplementation. While consistent fiber-specific trends were observed, the reduced sample size may have affected the overall robustness of the taxonomic and metabolic patterns identified, particularly for pectin, which exhibited donor-dependent responses. Future studies including larger and more diverse cohorts, as well as in vivo validation, will be important to confirm these findings and further evaluate the potential of multi-fiber strategies for microbiome modulation.

From a functional food perspective, these findings suggest that food formulations may benefit from incorporating complementary fibers of varying structural complexity rather than relying on a single prebiotic substrate. For example, combining β-glucans with structurally more complex fibers such as dextran and structurally simpler fibers such as inulin may simultaneously support Bacteroidota degraders and butyrate-producing Firmicutes while promoting the expansion of Actinobacteriota specialists. Such complementary microbial responses may improve the control of opportunistic Proteobacteria, broaden microbial diversity, and generate more balanced fermentative outputs than single-fiber formulations. Although these combinations require validation in larger in vitro studies and subsequent in vivo investigations, the present findings provide a framework for the future rational design of multi-fiber functional foods tailored to specific microbiome-related health outcomes.

## 4. Materials and Methods

### 4.1. Specimen Collection and In Vitro Fermentation

Fresh stool samples were obtained from two healthy donors (Age 18–35, BMI 18.5–23.9 kg/m^2^) recruited at Northwestern University (Evanston, IL, USA). Donors reported no gastrointestinal disease or antibiotic use within the previous three months and provided informed consent. The study was approved by the Northwestern University Institutional Review Board (IRB; protocol STU00206091), and all procedures were conducted in accordance with the approved guidelines. Samples were collected in sterile tubes, immediately frozen at −80 °C, and processed under anaerobic conditions at the Amato Lab (Evanston, IL, USA).

For slurry preparation, 2.5 g of fecal material from each donor was homogenized in 50 mL of PBS supplemented with 0.1% cysteine (PBSC), vortexed for 5 min, and filtered through four layers of sterile cheesecloth to obtain a 5% (*w*/*v*) fecal slurry.

Batch fermentations were performed following established protocols with minor modifications [112,113]. Briefly, 1 mL of filtrated fecal slurry was incubated with 19 mL of Gifu Anaerobic Broth (Sigma-Aldrich, St. Louis, MO, USA), which contains β-glucans and mannans (1.5 g/L, of yeast extract) [52,55,56,57], and dextrose (3 g/L) [114]. Substrates included inulin from chicory (Sigma-Aldrich, St. Louis, MO, USA; CAS No. 9005-80-5), pectin from citrus peel (Sigma-Aldrich, St. Louis, MO, USA; CAS No. P9135), and dextran NextDext™ from *Weissella cibaria* (AB Biotek HNH, Peterborough, UK), each added at 0.5 g per 50 mL, alongside a control without added fiber. Fermentations were carried out in sealed glass tubes at 37 °C with continuous stirring (150 rpm) for up to 48 h under strict anaerobic conditions.

Aliquots (1 mL for DNA and 1 mL for SCFA) were collected at baseline (0 h) and at 4, 8, 24, 32, and 48 h using a syringe by passing the needle through the rubber stopper to maintain anaerobic conditions. All collected samples were immediately frozen at −80 °C for downstream analyses. Duplicate incubations were performed for each treatment and donor, including paired controls.

### 4.2. Sample Processing for 16S rRNA Gene Amplicon Sequencing and SCFA Quantification

DNA was extracted from samples using the DNeasy Powersoil Pro Kit (Qiagen, Germantown, MD, USA) with modifications. After adding solution C1, samples were incubated at 65 °C for 10 min before vortexing for 10 min. The V4-V5 region of the 16S rRNA gene was amplified with the 515F/926R primers [115]. PCR products were purified and normalized using SequalPrep Normalization (Thermo Fisher Scientific, Waltham, MA, USA). Sequencing of barcoded amplicons was achieved on an Illumina MiSeq V3 platform (Illumina, San Diego, CA, USA) by the Rush Genomics and Microbiome core facility (Chicago, IL, USA) with a depth of at least 20,000 sequences per sample. DNA concentration and purity were assessed using standard quality control procedures to ensure adequate template quality for amplification. DNA extractions, PCR amplification and sequencing were performed as previously described by Mallot et al. [115].

Raw sequences were demultiplexed and quality filtered using the q2-demux plugin, followed by denoising with DADA2 [116] via the q2-dada2 implemented in the QIIME2 version 2020.6 pipeline [117]. Sequences from mitochondria and chloroplasts were removed. Amplicon sequence variants (ASVs) were identified with a learned sequencing error correction model (DADA2 method). The dataset contained an average of 10,429 reads per sample (range: 5070–17,805). Alpha-diversity metrics (Shannon diversity, observed ASVs, Faith’s phylogenetic diversity [118]), and beta diversity metrics (weighted UniFrac [119], unweighted UniFrac [120], Jaccard, Bray–Curtis [121,122]) were estimated using the q2-diversity plug-in after samples were rarefied to 5000 reads per sample. Taxonomy was assigned to ASVs using q2-feature-classifier [123] against Greengenes v.gg-13-8-99-nb-classifier [124] with a total of 2887 ASVs identified. DNA extraction and PCR negative controls were included to monitor contamination. Their taxonomic composition was compared to that of biological samples, and no contamination was detected; thus, negative controls were removed from subsequent analyses.

To determine fecal SCFA concentrations, SCFAs were extracted from 1 mL of fecal sample collected during the in vitro fiber fermentation, followed by 3-Nitrophenylhydrazine hydrochloride derivatization (3NPH) using the Han et al. 2015 chemical derivatization protocol [125]. Metabolites were measured using LC-MS/MS at the University of Illinois Chicago Proteomics Core. A stable isotope labeled 3NPH was used in internal standard derivatization. An Agilent 1290 UPLC (Agilent Technologies, Santa Clara, CA, USA) coupled to a QTRAP 5500 mass spectrometer (SCIEX, Framingham, MA, USA), which is equipped with Electrospray Ionization (ESI), was used for multiple reaction monitoring (MRM)-based quantification [126]. Quality control samples and internal standards were included throughout LC-MS/MS analysis to monitor instrument performance and ensure analytical reproducibility.

### 4.3. Statistical Analysis

Once processed, we used sequence data to compare richness, diversity, and microbial community composition among samples. We first tested the effect of the medium by comparing the baseline samples from 0 h to 4–8 h time points for the control with only yeast extract (β-glucans and mannans) to the same time points for the batches with added fibers. We then tested the effect of fiber enrichment by comparing the samples from each fermentation at 8 h to 24–32–48 h. Differences in fiber complexity were evaluated by comparing control samples containing only β-glucans and mannans with those supplemented with inulin, pectin, or dextran. All statistical analyses were performed in R (version 3.4.0). The QIIME outputs and associated metadata were imported into R as a phyloseq object using the QIIME2R package (version 0.99.6) [127]. For each sample type, differences in microbial richness and alpha diversity across fibers (Shannon, observed ASVs and Faith PD) were visualized using the phyloseq [128], ggplot2 [129], and ggsignif [130] packages in R and tested using Wilcoxon rank sum and Kruskal–Wallis tests (stats package; R software (version 3.4.0)). Differences in the microbial community composition in response to the different fibers were evaluated using unweighted and weighted UniFrac distance matrices. The significance of these patterns was assessed by permutational analysis of variance (PERMANOVA) using the adonis2 function in the vegan package [131]. Results were visualized using non-metric multidimensional scaling (NMDS) ordinations plots. Relative abundances of bacterial phyla across sample groups were visualized using bar plots in ggplot2. Differential abundance heatmaps based on the centered log-ratio (CLR) values were generated for bacterial families with relative abundances higher than 0.2% using the ComplexHeatmap package [132,133] with Aitchison distance dendrograms [134,135].

The relationships between the relative abundances of bacterial phyla in the fiber enrichment and the control samples were evaluated using a fixed effects linear regression model with the lme function in R. The differential abundance of bacterial at phylum, family and genus levels was assessed using analysis of composition of microbiomes with bias correction (ANCOM-BC) [136,137]. Given the limited sample size, we present both statistically significant results and noteworthy non-significant trends in our heatmaps.

To examine changes in short-chain fatty acid (SCFA) production during fermentation, concentrations of acetate, butyrate, propionate, and isovalerate were analyzed in R. Temporal trends were visualized with ggplot2, displaying both individual donor trajectories and mean concentration profiles across time points for each fiber treatment. Statistical analyses were conducted with the rstatix package using repeated-measures ANOVA to evaluate the effects of time and fiber type on SCFA production. Significant differences (*p* < 0.05) and notable temporal trends were reported. The resulting line plots illustrate both the reproducibility across donors and the distinct kinetics of SCFA accumulation driven by different fiber substrates.

## 5. Conclusions

Using anaerobic in vitro fermentation with human fecal inocula, we have demonstrated that structurally distinct dietary fibers drive substrate-specific ecological and metabolic restructuring of the gut microbiota. Although all treatments showed an early expansion of *Escherichia–Shigella* consistent with simple carbohydrate utilization, subsequent community trajectories diverged according to fiber structure. β-glucans/mannans established a strong acetate-producing baseline but showed incomplete suppression of opportunistic taxa and limited recovery of butyrate-associated consortia. Inulin/β-glucans/mannans elicited a canonical prebiotic response characterized by early Actinobacteriota activation followed by secondary butyrate engagement, resulting in coordinated SCFA production but failing to suppress Proteobacteria. Pectin/β-glucans/mannans stimulated butyrate-associated taxa but exhibited greater donor-dependent variability, highlighting the influence of baseline microbial composition on fiber responsiveness. Dextran/β-glucans/mannans induced the most coordinated restructuring, enriching *Bacteroides* and butyrate-producing Firmicutes while suppressing Proteobacteria, indicating enhanced cross-feeding stability and ecological control.

Together these findings show that fiber structure determines not only fermentative intensity but ecological directionality and reproducibility. Single fibers activate specific microbial groups, whereas structurally diverse substrates incite complementary degradative and cross-feeding networks. These results provide mechanistic support for multi-fiber dietary strategies aimed at enhancing butyrate production, suppressing pro-inflammatory taxa, and promoting microbiome resilience. Rather than treating “fiber” as a uniform intervention, future dietary interventions should consider structural diversity as a tool to fine-tune microbiome-driven metabolic outcomes, to restore microbial balance and support long-term host health. Understanding the differential ecological and metabolic signatures of individual fibers provides a rational framework for functional food design and precision-nutrition strategies aimed at modulating defined microbial pathways and controlling inflammation in chronic diseases like IBD.

## Data Availability

The raw sequence files are available at the NCBI Sequence Read Archive (https://www.ncbi.nlm.nih.gov/) under BioProject ID number PRJNA1475506.

## Supplementary Materials

The following supporting information can be downloaded at: https://www.mdpi.com/article/10.3390/molecules31152613/s1, Figure S1: Observed ASVs of the fecal microbial communities during in vitro fermentation across the control (β-glucans-mannans), dextran (control supplemented with dextran), inulin (control supplemented with inulin), and pectin (control supplemented with pectin) treatments at 0, 4, 8, 24, 32, 48 h; Figure S2: Faith PD of the fecal microbial communities during in vitro fermentation across the control (β-glucans-mannans), dextran (control supplemented with dextran), inulin (control supplemented with inulin), and pectin (control supplemented with pectin) treatments at 0, 4, 8, 24, 32, 48 h.
