# Supplementary material for: How Complex Dietary Fibers Can Be Used to Shape the Human Gut Microbiome Toward Reduced Inflammatory Potential: A Pilot Study
Source: Molecules. 2026 Jul 27;31(15):2613. doi: 10.3390/molecules31152613 (PMC13468651; doi:10.3390/molecules31152613)
Supplement: Supplementary file 1 [file molecules-31-02613-s001.zip › molecules-4412700-supplementary.pdf]

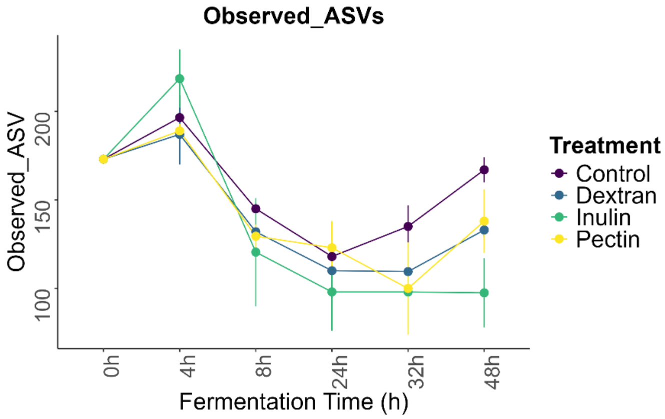

Figure S1. Observed ASVs of the fecal microbial communities during in vitro fermentation across the Control (β-glucans-mannans), dextran (Control supplemented with dextran), inulin (Control supplemented with inulin), and pectin (Control supplemented with pectin) treatments at 0, 4, 8, 24, 32, 48 h.

.

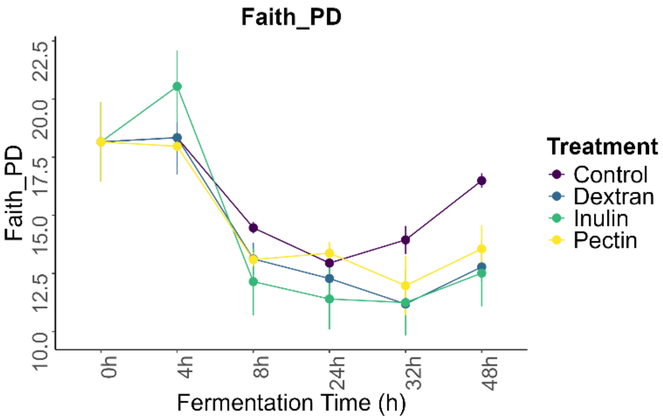

Figure S2. Faith PD of the fecal microbial communities during in vitro fermentation across the Control (β-glucans-mannans), dextran (Control supplemented with dextran), inulin (Control supplemented with inulin), and pectin (Control supplemented with pectin) treatments at 0, 4, 8, 24, 32, 48 h.
